# Experimental periodontitis induced hypoadiponectinemia by IRE1α-mediated endoplasmic reticulum stress in adipocytes

**DOI:** 10.1186/s12903-023-03758-6

**Published:** 2023-12-21

**Authors:** Qianqi Wu, Li Yan, Xiao Wu, Yiyan Chen, Leilei Ye, Yingtao Lv, Yuan Su

**Affiliations:** 1 Stomatology Center, Shunde Hospital, Southern Medical University, The First People’s Hospital of Shunde, NO.1 Jiazi Road, Foshan, 528300 Guangdong China; 2 Department of Periodontology, Stomatological Hospital, Southern Medical University, Guangzhou, China; 3 Department of Implantology and Prosthodontics, Stomatological Hospital, Southern Medical University, Guangzhou, China

**Keywords:** Periodontitis, Endoplasmic reticulum stress, Hypoadiponectinemia, GRP78, IRE1α

## Abstract

**Backgroud:**

Hypoadiponectinemia is the important cause of insulin resistance. Recent studies have shown that periodontitis is associated with hypoadiponectinemia. The purpose of this study was to investigate the effect of periodontitis-induced endoplasmic reticulum stress (ERS) in visceral adipocytes on hypoadiponectinemia.

**Methods:**

Rat periodontitis models were established by local ligation with silk around the bilateral maxillary second molars. *Porphyromonas gingivalis-*lipopolysaccharid (*P.g*-LPS) was also used to stimulate the visceral adipocytes in vitro. The protein expression levels of glucose regulated protein 78 (GRP78), inositol-requiring protein 1α (IRE1α), protein kinase RNA-like ER kinase (PERK), activating transcription factor 6 (ATF6) and adiponectin were detected. IRE1α lentiviruses were transfected into visceral adipocytes in vitro, and an IRE1α inhibitor (KIRA6) was injected in epididymal adipose tissue of rats to detect and verify the effect of ERS on adiponectin expression in visceral adipocytes in vivo.

**Results:**

Hypoadiponectinemia was observed in periodontitis rat, and the expression levels of ERS key proteins GRP78 and the phosphorylation levels of IRE1α (p-IRE1α)/IRE1α in visceral adipocytes were increased, while the expression levels of adiponectin protein were decreased. After KIRA6 injection into epididymal adipose tissue of rats with periodontitis, adiponectin levels in visceral adipocytes increased, and serum adiponectin levels recovered to a certain extent. The protein expression levels of GRP78 and p-IRE1α/IRE1α were increased and adiponectin protein expression was decreased in *P.g*-LPS-induced visceral adipocytes. Overexpression of IRE1α further inhibited adiponectin expression in *P.g*-LPS-stimulated visceral adipocytes, and conversely, IRE1α inhibition restored adiponectin expression.

**Conclusions:**

Our findings suggest that periodontitis induces ERS in visceral adipocytes leading to hypoadiponectinemia. IRE1α is a key protein regulating adiponectin expression in visceral adipocytes.

**Supplementary Information:**

The online version contains supplementary material available at 10.1186/s12903-023-03758-6.

## Introduction

Adiponectin is a 30-kDa protein produced mainly by visceral adipocytes and secreted into the peripheral blood [1]. Circulating adiponectin concentrations can reach 2–20 µg/mL, accounting for 0.01–0.05% of total serum protein [2]. Circulating adiponectin can reach many tissues, such as the heart, liver, kidney, pancreas, and skeletal muscle [3]. It binds to adiponectin receptors on the surface of target cells and plays an active anti-inflammatory, anti-atherosclerotic and anti-diabetic role [4]. Thus, homeostasis of plasma adiponectin levels is essential for systemic health. Recent studies have confirmed that low serum adiponectin levels, namely, hypoadiponectinemia, are associated with the development and poor prognosis of reproductive, digestive, endocrine, cardiovascular, and other systemic diseases. For example, low serum adiponectin levels may promote the occurrence and development of endometrial cancer (OR: 10.64, 95% CI: 3.61–31.40) [5], prostate cancer (HR: 2.44; 95% CI: 1.57–3.79) [6], and breast cancer (HR: 3.75; 95% CI: 1.37–10.25) [7]. Long-term hypoadiponectinemia is associated with an increased risk of colorectal cancer (OR: 2.83, 95% CI: 1.50–5.34) [8]. Numerous recent studies have confirmed that hypoadiponectinemia is an important cause of insulin resistance [9–11]. Insulin resistance refers to the decreased sensitivity of insulin target tissue (adipose tissue, skeletal muscle, liver) to insulin, which is an important pathological change in prediabetes [12]. If insulin resistance is not effectively alleviated, it will eventually lead to the development of type 2 diabetes (T2D) [13]. Therefore, it is of great clinical significance to reveal the pathogenesis of hypoadiponectinemia and improve the serum adiponectin level to prevent and treat associated systemic diseases, especially insulin resistance and T2D [14, 15].

Epidemiological investigations have found that hypoadiponectinemia is associated with periodontitis [16, 17]. Periodontitis is a lasting inflammatory disease that occurs in the oral cavity. Subgingival microbial dysbiosis is an essential pathological feature [18]. Disordered subgingival flora leads to a marked increase in pathogenic microorganisms and toxic products, causing inflammation and destructive absorption of periodontal supporting tissues, such as gingiva, periodontal ligaments, and alveolar bones, from multiple or even all teeth [19]. According to the latest report, the global prevalence of periodontitis is as high as 50%, among which severe periodontitis affects 1.1 billion people [20]. In patients with periodontitis, the serum adiponectin level was significantly lower than that of the periodontal healthy population, which may contribute to the development of hypoadiponectinemia-related diseases [17]. However, how periodontitis causes hypoadiponectinemia has not been reported. Pathogenic microorganisms and toxic products in patients with periodontitis may enter the systemic circulation from the epithelial tissue ulcer in the periodontal pocket and lead to bacteraemia, endotoxaemia, or colonization of other tissues far from the mouth [21]. These virulence factors induce or aggravate the immune and inflammatory responses or metabolic dysfunction of the tissues [22, 23]. LPS secreted by *Porphyromonas gingivalis* (*P.g-*LPS) is a component of the outer wall of bacteria. Previous studies have shown that *P.g-*LPS is one of the key virulence factors in the negative effects of periodontitis on systemic health [24] *P.g-*LPS caused local periodontal destruction but also caused endotoxaemia and mediated pathologic changes in liver and visceral adipose tissue [25–27]. Visceral adipose tissue is a crucial site for synthesizing and secreting adiponectin [2]. Any reason, such as genetic, environmental, and other factors leading to the disorder of adiponectin synthesis and secretion in visceral adipocytes, is an important cause of hypoadiponectinemia [28]. Studies have found that periodontitis can lead to abnormal secretion of visceral adipocytes, specifically increased secretion of the adipokines interleukin-6 (IL-6), tumour necrosis factor-α (TNF-α) and leptin and decreased secretion of adiponectin [29]. Further studies showed that *P.g-*LPS could mediate the abnormal secretion function of visceral adipocytes in vitro, as evidenced by increased leptin and decreased adiponectin expression [30]. However, whether *P.g-*LPS-induced periodontitis can induce hypoadiponectinemia in vivo and the related mechanisms have not been reported.

Adipokine assembly and secretion are closely related to the endoplasmic reticulum (ER) function of visceral adipocytes. Under pathological stimuli, such as hypoxia and inflammation, intracellular protein folding is impaired, resulting in misfolding or accumulation of unfolded proteins. The ER responds to pressure by activating a series of signals to reduce the accumulation of unfolded proteins and maintain protein homeostasis, which is called endoplasmic reticulum stress (ERS). ERS in visceral adipocytes can alter the synthesis and secretion of specific adipokines. ERS participates in the regulation of cellular physiology and pathology by activating three signalling pathways mediated by inositol-requiring protein 1α (IRE1α), protein kinase RNA-like ER kinase (PERK), and activating transcription factor 6 (ATF6) [31]. Among them, the ERS pathway mediated by IRE1α was confirmed to be related to adiponectin secretion in visceral adipocytes [32]. Studies have found that the increased expression of glucose regulated protein 78 (GRP78) and IRE1α in visceral adipose tissue of obese mice resulted in decreased adiponectin levels [33]. Conversely, in the study by Cho et al., [34] activation of IRE1α in 3T3-L1 adipocytes resulted in increased adiponectin levels in response to insulin stimulation. These results suggest that IRE1α is a key protein that regulates adiponectin secretion in visceral adipocytes under ERS, but its regulation of adiponectin expression is entirely different under different stimuli. However, it is not clear whether ERS occurs in rat visceral adipose tissue in the context of periodontitis and whether its marker protein IRE1α is a key molecule that regulates adiponectin expression and causes hypoadiponectinemia in rats.

Thus, the periodontitis model of rats was established by periodontal ligation and a *P.g-*LPS-stimulated visceral adipocytes model in vitro, and the association of IRE1α-mediated ERS with hypoadiponectinemia was explored to preliminarily reveal the underlying mechanism behind hypoadiponectinemia caused by periodontitis in this study.

## Materials and methods

### Establishment of the rat periodontitis model

A total of 18 male Sprague‒Dawley (SD) rats, aged 8 weeks and weighing approximately 250–300 g each, were kept in cages at a temperature of 23 ± 2 °C under an appropriate 12-hour light-dark cycle. Animals were randomly divided into three groups (n = 6): control group, periodontitis group, and periodontitis + KIRA6 (IRE1α inhibitor) group. In the periodontitis group, 1.5% pentobarbital sodium was used under intraperitoneal anesthesia (30 mg/kg) to tie at the cervical region of the bilateral maxillary second molars with 5.0 silk. The control group was not treated [35]. The rats in the periodontitis + KIRA6 group was also subjected to periodontal ligature, and a dose of 10 mg/kg KIRA6 (MCE, New Jersey, USA) according to the study by Yi et al., [36] was injected into epididymal adipose tissue at the same time. The silk ligatures of each rat were checked and KIRA6 was injected once a week under anesthesia. After 3 months, rats were also fasted for 12 h prior to experiments and anesthetized (50 mg/kg, intraperitoneal) with 1.5% pentobarbital sodium. Experiments were performed 10–15 min later. Median laparotomies were performed in the rats, and blood samples were taken via inferior vena cava punctures and distributed into tubes containing heparin. Plasma was prepared by centrifuging the blood at 3000 rpm for 15 min at 4 °C and was stored in aliquots at -80 °C for later blood biochemical parameter analyses. Samples of the bilateral maxillary molar regions and epididymal adipose tissue were removed and fixed in 4% paraformaldehyde.

### Analysis of alveolar bone loss

Maxillary bones were scanned by using micro-CT (sky1172, Bruker, Germany). Three-dimensional reconstruction was performed for each sample. Total alveolar bone volume (TV) and bone volume (BV) were measured, and the bone volume fraction (BVF = BV/TV) was calculated [37]. These parameters were used to analyse the alveolar bone loss of bilateral maxillary second molars.

### HE staining of periodontal tissues

The maxillary samples were decalcified with 10% tetrasodium-EDTA aqueous solution (pH 7.0) for 12 weeks at 4 °C. The periodontal tissue samples were embedded in paraffin, and sections (4 μm thick) were stained with haematoxylin and eosin (HE). A histological analysis of the site with induced periodontitis was carried out to observe the changes in these tissues. These digital images were taken using an optical microscope (DM4B, LEICA, Germany).

### Detection of serum adiponectin, fasting plasma glucose and fasting plasma insulin

Serum was collected, and adiponectin, fasting plasma glucose and fasting plasma insulin were detected using an enzyme-linked immunosorbent assay (ELISA) kit (Cikebio, Beijing, China). All assays were performed following the manufacturer’s instructions.

### Quantitative real-time polymerase chain reaction (qRT‒PCR)

Total RNA was extracted from epididymal adipose tissue using TRIzol reagent (Invitrogen, CA, USA) according to the manufacturer’s instructions. RNA quantity and quality were measured by a BioPhotometer Plus photometer (Eppendorf, Hamburg, Germany). Approximately 1 µg of total RNA was used for cDNA synthesis, and then the resulting cDNA was used as a template for PCR amplification. Gene expression was subsequently determined in triplicate using SYBR Green qPCR SuperMix (Invitrogen, CA, USA) and the ABI PRISM^®^ Real-Time PCR Sequence Detection System. Cycling protocols were performed under the following conditions: initial denaturation at 95 °C for 5 min, followed by 40 cycles of denaturation at 95 °C for 15 s and annealing at 60 °C for 32 s. The relative gene expression was determined with the 2^-ΔΔCt^ method using β-actin as an endogenous control. The designed primers are listed in Table 1.

**Table 1:** Primer sequences of genes for quantitative real time-PCR

Gene	Forward primer	Reverse primer
Adiponectin	5‘- TGTTCTTGGTCCTAAGGGTGAC-3’	5‘- CCTACGCTGAATGCTGAGTGA-3’
GRP78	5‘- GACTGGAATCCCTCCTGCTC-3’	5‘- GGTCAGGCGGTTTTGGT-3’
IRE1α	5‘- GGAGACCCTACGCTATTTGACCT-3’	5‘- TTGCTGACAATCTTGAGGGAG-3’
PERK	5‘- CCAGTTTTGTACTCCAATTGCA-3’	5‘- CAGATACAGCTGGCCTCTATAC-3’
ATF6	5‘- TTACTCACCGATCCGAGTTG-3’	5‘- AAACTGGACATCATCCGTGT-3’
β-actin	5‘- CCCATCTATGAGGGTTACGC-3’	5‘- TTTAATGTCACGCACGATTTC-3’

### Western blotting analysis

Total protein samples were extracted from the epididymal adipose tissue and adipocytes using lysis buffer (Beyotime, Shanghai, China) containing a PMSF protease inhibitor, and the protein concentrations were determined by a BCA™ protein assay. Approximately 20 µg of total protein was loaded and separated in 8% SDS-polyacrylamide gels. The membrane was blocked in 5% nonfat milk on a shaker for 1 h and incubated overnight at 4 °C with the following primary antibodies: anti-GRP78 (1:1000 dilution; Abcam, Cambridge, UK), anti-IRE1α (1:1000 dilution; Abcam, Cambridge, UK), anti-p-IRE1α (1:1000 dilution; Abcam, Cambridge, UK), anti-adiponectin (1:1000 dilution; Abcam, Cambridge, UK), anti-PERK (1:1000 dilution; Abcam, Cambridge, UK), anti-p-PERK (1:1000 dilution; Abcam, Cambridge, UK), anti-ATF6 (1:1000 dilution; Abcam, Cambridge, UK), and anti-β-actin antibody (1:1000 dilution; Abcam, Cambridge, UK). After the washing procedure with Tris-buffered saline with Tween, the membranes were incubated with secondary antibodies on a shaker for 50 min at room temperature. A chemiluminescence imager detected the protein intensity, and the protein amounts were analysed using the ImageJ program. In order to reduce non-specific protein expression during the experiment, the membrane was trimmed according to the protein size range provided in the antibody instructions prior to hybridization with the antibody. The original images were shown in Supplementary File. 1.

### Cell cultures

Adipocytes were isolated from the epididymal adipose tissue of SD rats. They were seeded in sterile T25 culture flasks at equal densities with 5 mL of Dulbecco’s minimal essential medium (Gibco, Waltham, USA) supplemented with 20% foetal bovine serum (Gibco, Waltham, USA) and 1% penicillin/streptomycin (Gibco, Waltham, USA) at 37 °C in a humidified atmosphere of 5% CO_2_. The cell culture medium was replaced every other day. The adipocytes were used for experiments between the 4th and 5th passages.

### Oil red o stain

The cells were fixed with 10% formalin for 10–15 min at room temperature and then stained with Oil Red O (Jiancheng, Nanjing, China) solution for 15 min. The lipid droplets were observed using an optical microscope (DM4B, LEICA, Germany).

### CCK8 experiment

The 4th passage of adipocytes was seeded in a 96-well culture plate at a density of 5 × 10^4^ cells on each plate in 100 µL culture medium. According to our previous studies, the addition of 100 ng/mL *P.g-*LPS (InvivoGen, San Diego, USA) to visceral adipocytes for 4 h induced changes in adiponectin secretion function. Therefore, the cells were incubated for 24 h at 37 °C in a 5% CO_2_ incubator, followed by the addition of 100 ng/mL *P.g-*LPS for 4 and 8 h. Then, 10 µL of CCK-8 solution was added to each well and incubated for 4 h. Each well’s optical density (OD) value was measured at a wavelength of 450 nm using a microplate reader (Thermo^®^, Germany).

### Models of IRE1α overexpression and inhibition in visceral adipocytes

The pLVX-IRE1α lentiviral vector was utilized to construct the pLVX-IRE1α plasmid and pLVX-IRE1α-RNAi plasmid. Moreover, negative sham oligonucleotides for IRE1α (pLVX-NC) were also synthesized by JiYuan BioTech (Guangzhou, China). After 24 h of incubation, the cells were supplemented with fresh serum-free medium. Then, adipocytes were treated with puromycin (2 µg/mL 5–7 days; Solarbio, Beijing, China) for stable transfection.

### Statistical analysis

Differences between the two groups were assessed by independent sample *t* test, and differences among multiple groups were evaluated by one-way ANOVA. The threshold for statistical significance was set at *P* < 0.05 The correlations between adiponectin and HOMA-IR were analysed using Pearson’s correlation analysis. All analyses were performed using SPSS 13.0.

## Results

### Local periodontal ligation induced periodontitis in rats

**Fig. 1:**
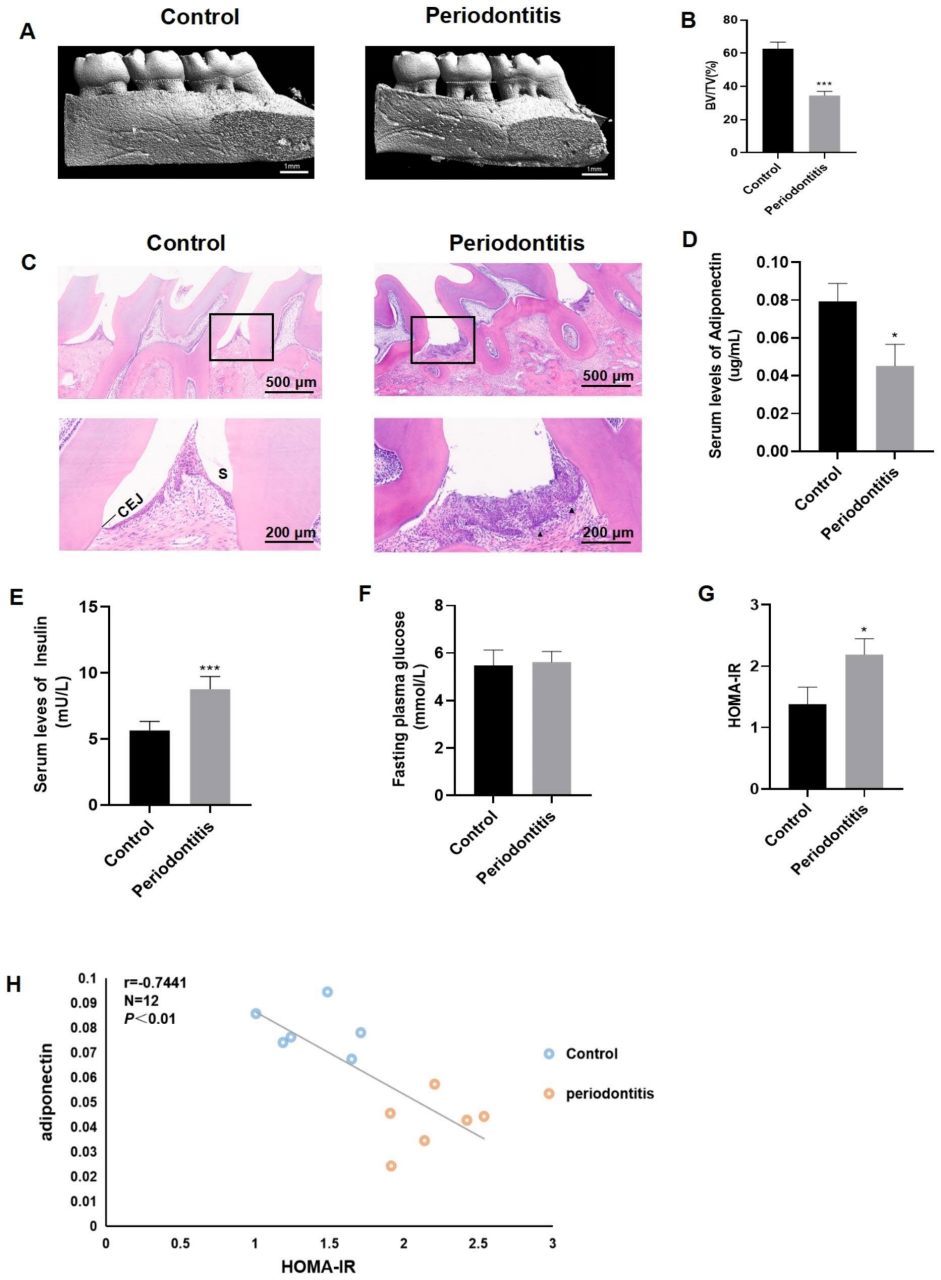
**Hypoadiponectinemia and insulin resistance induced by periodontitis in rats**. (**A**) Micro-CT images show the representative three-dimensional contours of alveolar bone in the control group and the periodontitis group. (**B**) The BV/TV value of the maxilla in periodontitis was significantly decreased. (**C**) HE staining shows the pathological changes in rat periodontal tissue. The control group (scale bar, 500 μm) with a black box designating close-up (scale bar, 200 μm) shows a normal periodontal structure. The periodontitis group (scale bar, 500 μm) with a black box designating close-up (scale bar, 200 μm) shows junctional epithelium migration to the roots, and inflammatory cells infiltrate the connective tissue (black arrowheads). (**D**) Serum adiponectin level in the periodontitis group was significantly lower than that in the control group. (**E**) Serum level of fasting plasma insulin was significantly higher in the periodontitis group. (**F**) Fasting plasma glucose level was not different between the two groups. (**G**) The HOMA-IR in the periodontitis group was higher than that in the control group. (**H**) Adiponectin was inversely associated with HOMA-IR. CEJ = cement-enamel junction, S = sulcus, Data are presented as the mean ± SD. *P < 0.05, ***P < 0.001 vs. the control group

During the experiment, the silk ligatures of rats did not loosen. Micro-CT images show the representative three-dimensional contours of alveolar bone in the control group and in the periodontitis group (Fig. 1A). Compared with the control group, the BV/TV value of the maxilla in the periodontitis group was significantly decreased (*P* < 0.001) (Fig. 1B). HE-stained sections of periodontal tissue from the control group revealed normal periodontal structures. In contrast, the HE-stained sections from the periodontitis group showed migration of connective epithelium into roots and infiltration of inflammatory cells into connective tissue (Fig. 1C). These results indicated that local periodontal ligation successfully induced the periodontitis model in rats.

### Hypoadiponectinemia and insulin resistance induced by periodontitis in rats

The serum level of adiponectin in the periodontitis group was decreased compared with that in the control group (*P* < 0.05) (Fig. 1D), indicating that periodontitis induced hypoadiponectinemia. Compared with the control group, the serum fasting plasma insulin level in the periodontitis group was significantly higher (*P* < 0.001) (Fig. 1E). However, there was no difference in fasting plasma glucose between the two groups (Fig. 1F). The formula fasting plasma glucose×fasting plasma insulin/22.5 was used to calculate homeostatic model assessment of insulin resistance (HOMA-IR). The HOMA-IR in the periodontitis group was higher than that in the control group (*P* < 0.05) (Fig. 1G). Serum adiponectin level was inversely related to HOMA-IR (Fig. 1H). The results suggested that hypoadiponectinemia may promote insulin resistance.

### Periodontitis induced ERS and decreased the expression of adiponectin in rat visceral adipose tissue

**Fig. 2:**
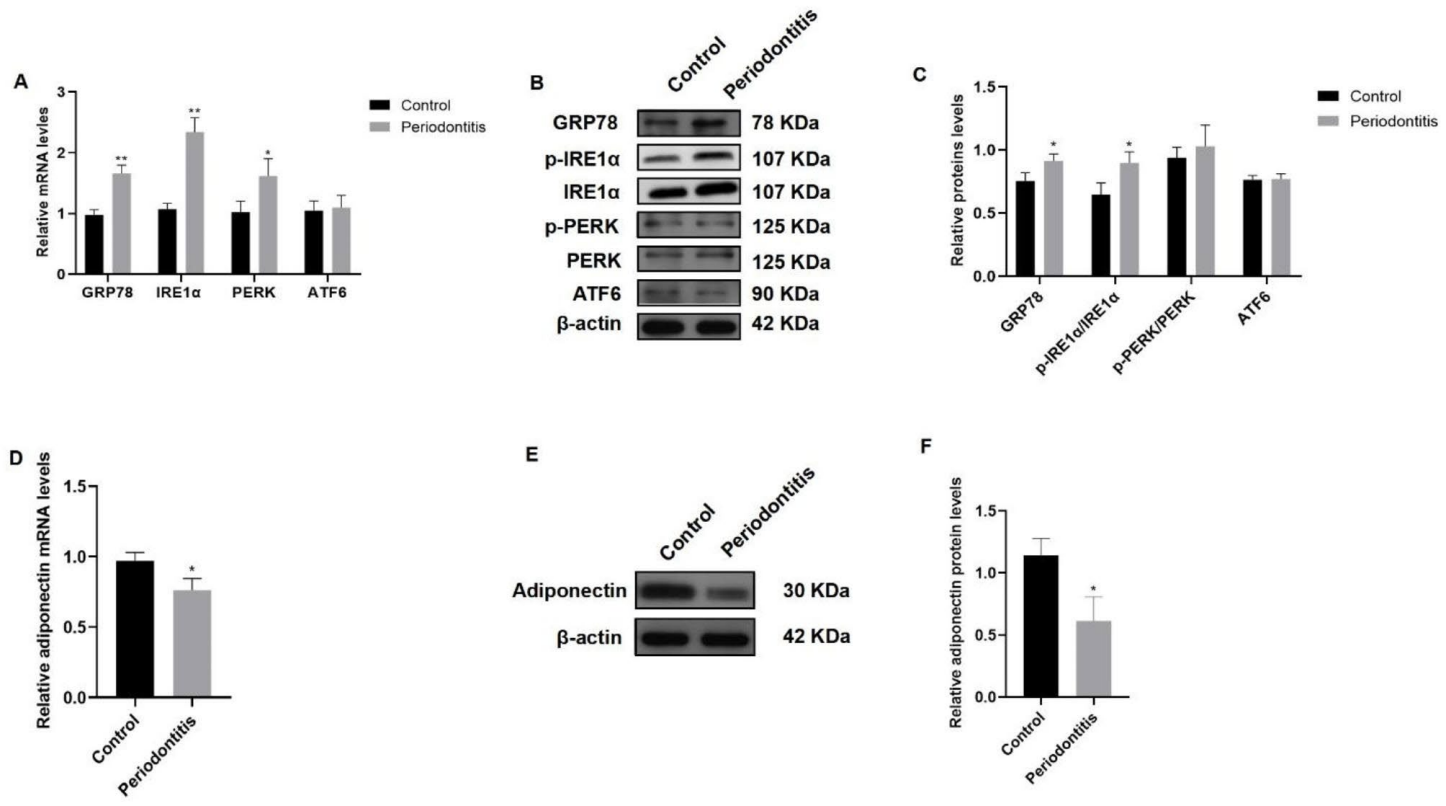
**Periodontitis induced ERS and decreased the expression of adiponectin in rat visceral adipocytes.** (**A**) The mRNA levels of GRP78, IRE1α and PERK in the periodontitis group were significantly higher than those in the control group, but ATF6 did not change. (**B**) Western blotting image of GRP78, IRE1α, p-IRE1α, PERK, p-PERK and ATF6 expression. (**C**) GRP78 and p-IRE1α/IRE1α expression was significantly higher than that in the control group, but p-PERK/PERK and ATF6 expression was not altered. (**D**) The mRNA level of adiponectin in the periodontitis group was lower than that in the control group. (**E**) Western blotting image of adiponectin expression. (**F**) Adiponectin expression was lower than that in the control group. The samples derive from the same experiment and that blots were processed in parallel. The full-length blots are presented in Supplementary File 1. Fig. 1S. GRP78 = glucose-regulated protein 78; IRE1α = inositol-requiring protein 1α; p-IRE1α = phosphorylation levels of IRE1α; PERK = double-stranded RNA-dependent protein kinase (PKR)-like ER kinase; p-PERK = phosphorylation levels of PERK; ATF6 = activating transcription factor 6. Data are presented as the mean ± SD. *P < 0.05, **P < 0.01 vs. the control group

qRT‒PCR analysis showed that the expression of GRP78, IRE1α and PERK in the periodontitis group was higher than that in the control group (*P* < 0.05), but ATF6 did not change (Fig. 2A). The protein levels of GRP78, IRE1α, the phosphorylation levels of IRE1α (p-IRE1α), PERK, the phosphorylation levels of PERK (p-PERK) and ATF6 were measured by western blotting (Fig. 2B). Compared with the control group, the periodontitis group exhibited higher expression of GRP78 and p-IRE1α/IRE1α (*P* < 0.05), but not p-PERK/PERK and ATF6 (Fig. 2C). These results indicated that ERS occurred in visceral adipose tissue of the periodontitis group. Additionally, we measured adiponectin expression in visceral adipose tissue. At the mRNA level, the expression of adiponectin was downregulated in the periodontitis group compared with the control group (*P* < 0.05) (Fig. 2D). The protein level of adiponectin was detected by western blotting (Fig. 2E). Adiponectin expression was significantly decreased in the periodontitis group compared with the control group (*P* < 0.05) (Fig. 2F).

### Identification of visceral adipocytes and CCK8 assay

**Fig. 3:**
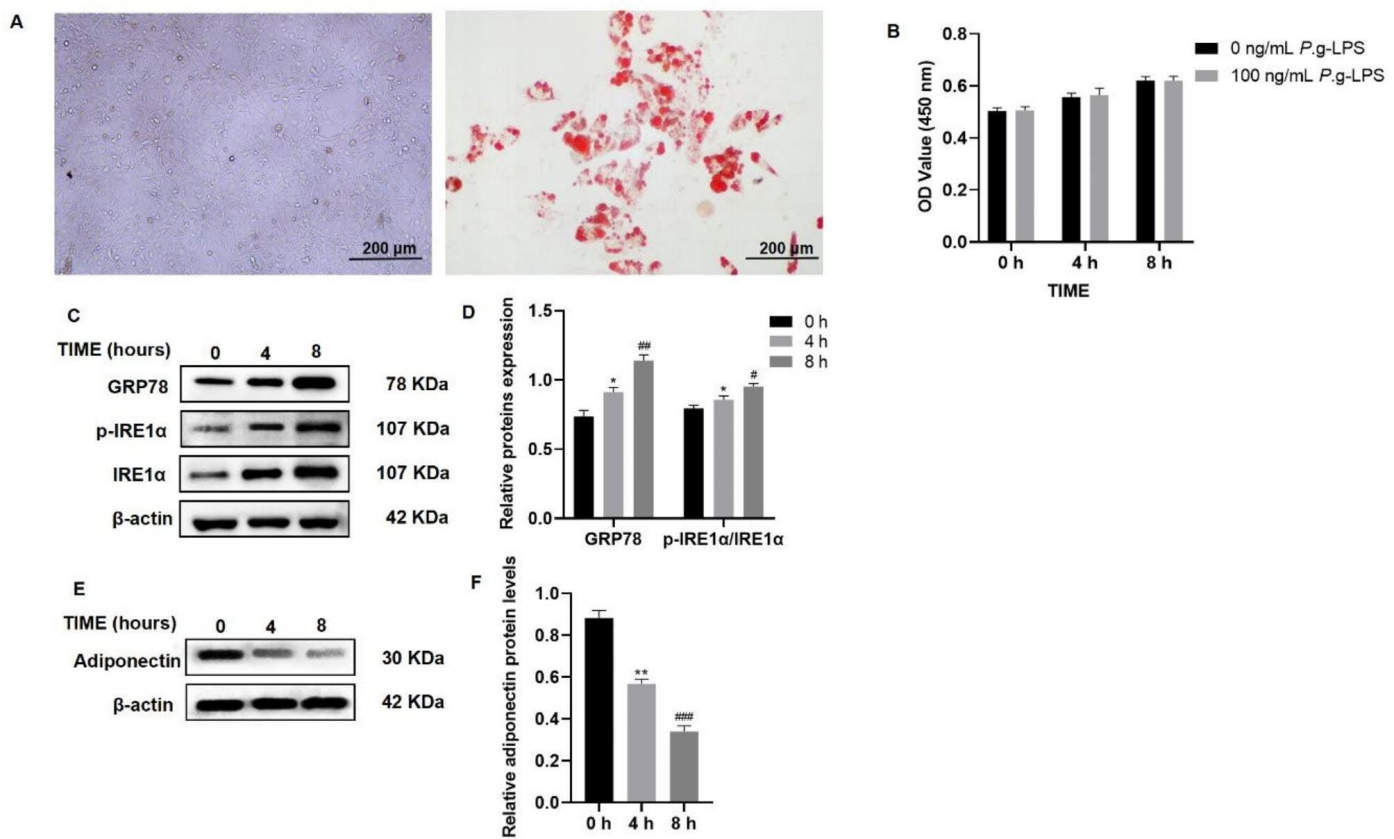
***P.g*-LPS induced ERS and downregulated adiponectin expression in visceral adipocytes in vitro.** (**A**) Optical microscope images show the culture of visceral adipocytes and Oil Red O staining for identifying adipocytes (scale bar, 200 μm). (**B**) CCK8 analysis of *P.g*-LPS concentration on the proliferation of visceral adipocytes. (**C**) Western blotting image of GRP78, IRE1α, and p-IRE1α expression in visceral adipocytes treated with 100 ng/mL *P.g*-LPS for 0, 4, and 8 h. (**D**) GRP78 and p-IRE1α/IRE1α expression was upregulated in a time-dependent manner. (**E**) Western blotting image of adiponectin expression in visceral adipocytes treated with 100 ng/mL *P.g-*LPS for 0, 4, and 8 h. (**F**) Adiponectin expression significantly decreased in a time-dependent manner. The samples derive from the same experiment and that blots were processed in parallel. The full-length blots are presented in Supplementary File 1. Fig. 2S. Data are presented as the mean ± SD. *P < 0.05, **P < 0.01 vs. the 0 h group, #*P* < 0.05, ##*P* < 0.01, ###*P* < 0.001 vs. the 4 h group

Visceral adipocytes differentiation was evaluated by Oil Red O staining. After 2 weeks of isolation and culture, the cellular morphology of visceral adipocytes was observed using an optical microscope and Oil Red O staining indicated the presence of fat globules (Fig. 3A). It confirmed that isolated and cultured visceral adipocytes differentiate into mature adipocytes.

Adipocytes were stimulated with 100 ng/mL *P.g-*LPS for 0, 4 and 8 h, and the activity of adipocytes were detected by CCK8 assay. The results showed that *P.g*-LPS had no effect on the viability of adipocytes (Fig. 3B).

### *P.g-*LPS induced ERS and downregulated adiponectin expression in visceral adipocytes in vitro

100 ng/mL *P.g-*LPS was used to stimulate visceral adipocytes for 0, 4, and 8 h. The expression of GRP78, IRE1α and p-IRE1α was detected by western blotting (Fig. 3C). The expression of GRP78 and p-IRE1α/IRE1α was upregulated in a time-dependent manner (Fig. 3D). These results indicated that ERS occurred in visceral adipocytes stimulated with *P.g-*LPS. The expression of adiponectin was detected by western blotting (Fig. 3E). The analysis revealed that adiponectin expression significantly decreased in a time-dependent manner (Fig. 3F). Four hours of stimulation with *P.g*-LPS induced ERS and significant downregulation of adiponectin expression in visceral adipocytes. Therefore, this processing time was used for subsequent experiments.

### IRE1α overexpression and silencing changed *P.g*-LPS-induced adiponectin expression levels in visceral adipocytes

**Fig. 4:**
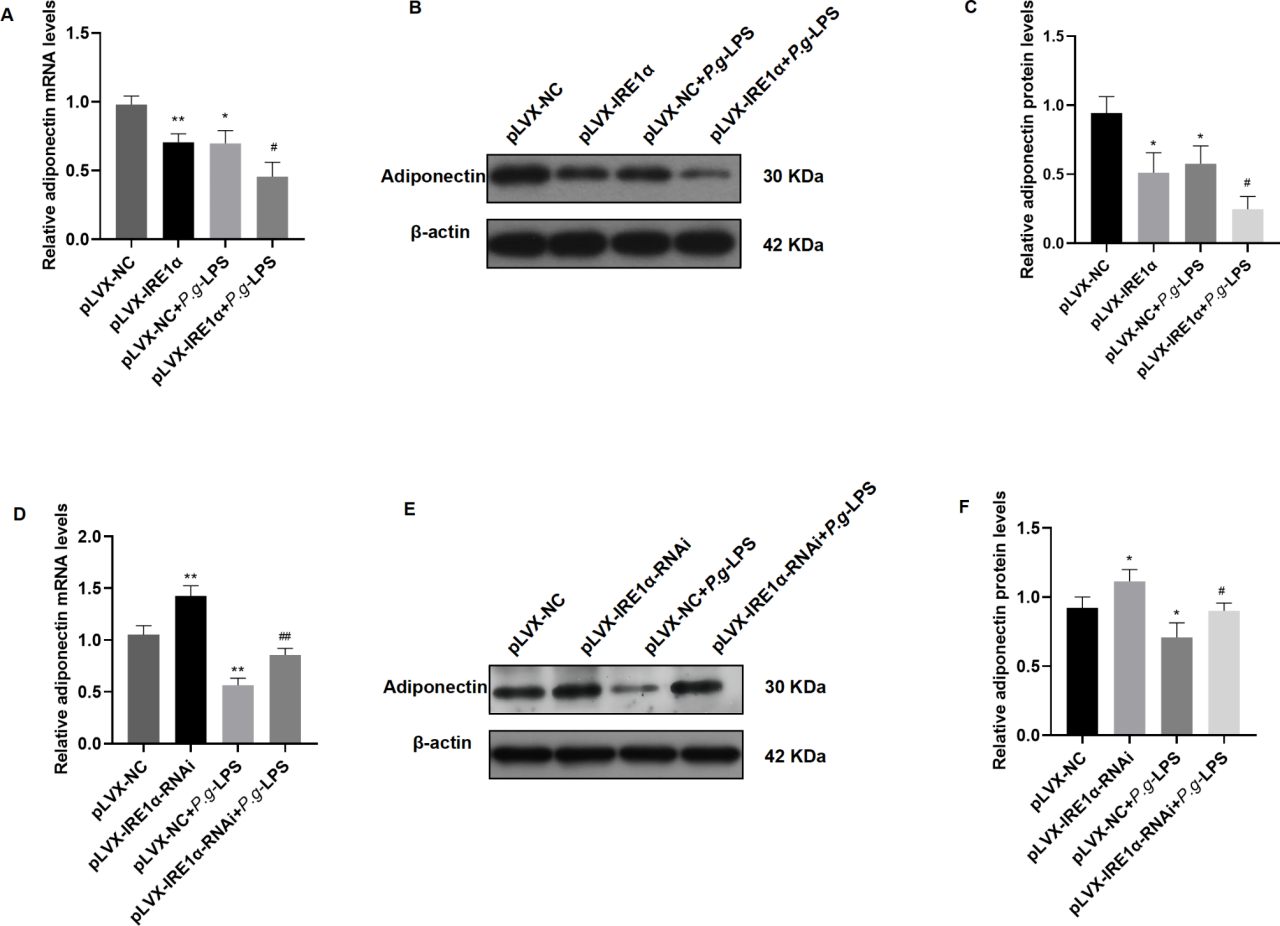
**Effect of IRE1α overexpression and inhibition on adiponectin expression in visceral adipocytes.** (**A**) The mRNA level of adiponectin in the pLVX-IRE1α group was lower than that in the pLVX-NC group. The adiponectin mRNA level in the pLVX-IRE1α + *P.g*-LPS group was significantly decreased compared to that in the pLVX-NC + *P.g*-LPS group. (**B**) Western blotting image of adiponectin expression. (**C**) Adiponectin expression in the pLVX-IRE1α group was lower than that in the pLVX-NC group. The expression of adiponectin in the pLVX-IRE1α + *P.g*-LPS group was decreased compared to that in the pLVX-NC + *P.g*-LPS group. (**D**) Compared with the pLVX-NC group, the mRNA level of adiponectin was increased in the pLVX-IRE1α-RNAi group. The adiponectin mRNA level in the pLVX-IRE1α-RNAi + *P.g*-LPS group was higher than that in the pLVX-NC + *P.g*-LPS group. (**E**) Western blotting image of adiponectin expression. (**F**) Adiponectin expression was increased in the pLVX-IRE1α-RNAi group compared with the pLVX-NC group. The expression of adiponectin in the pLVX-IRE1α-RNAi + *P.g-*LPS group was higher than that in the pLVX-NC + *P.g*-LPS group. The samples derive from the same experiment and that blots were processed in parallel. The full-length blots are presented in Supplementary File. 1 Fig. 3S. Data are presented as the mean ± SD. *P < 0.05, **P < 0.01 vs. the pLVX-NC group, #*P* < 0.05, ##*P* < 0.01 vs. the pLVX-NC + *Pg-*LPS group

After transfection with pLVX-IRE1α and pLVX-IRE1α-RNAi, 100 ng/ml *P.g-*LPS was used to stimulate visceral adipocytes for 4 h. qRT‒PCR analysis showed that compared with the pLVX-NC group, the level of adiponectin was decreased in the pLVX-IRE1α group (*P* < 0.01). The adiponectin level was downregulated in the pLVX-IRE1α + *P.g-*LPS group compared to the pLVX-NC + *P.g-*LPS group (*P* < 0.05) (Fig. 4A). The protein level of adiponectin was measured by western blotting (Fig. 4B). Adiponectin expression in the pLVX-IRE1α group was reduced compared with that in the pLVX-NC group (*P* < 0.05). The expression of adiponectin was significantly decreased in the pLVX-IRE1α + *P.g-*LPS group compared to the pLVX-NC + *P.g-*LPS group *(P* < 0.05) (Fig. 4C).

On the other hand, the mRNA level of adiponectin was significantly upregulated in the pLVX-IRE1α-RNAi group compared with the pLVX-NC group (*P* < 0.01). When compared with the pLVX-NC + *P.g-*LPS group, the adiponectin level in the pLVX-IRE1α-RNAi + *P.g-*LPS group was increased (*P <* 0.01) (Fig. 4D). The protein level of adiponectin was detected by western blotting (Fig. 4E). Adiponectin expression was increased in the pLVX-IRE1α-RNAi group compared with the pLVX-NC group (*P* < 0.05). The expression of adiponectin in the pLVX-IRE1α-RNAi + *P.g-*LPS group was higher than that in the pLVX-NC + *P.g-*LPS group (*P* < 0.05) (Fig. 4F). These findings indicated that IRE1α could regulate the expression of adiponectin in visceral adipocytes stimulated with *P.g-*LPS.

### Epididymal adipose tissue injection of KIRA6 improved hypoadiponectinemia and insulin resistance in rats with periodontitis

**Fig. 5:**
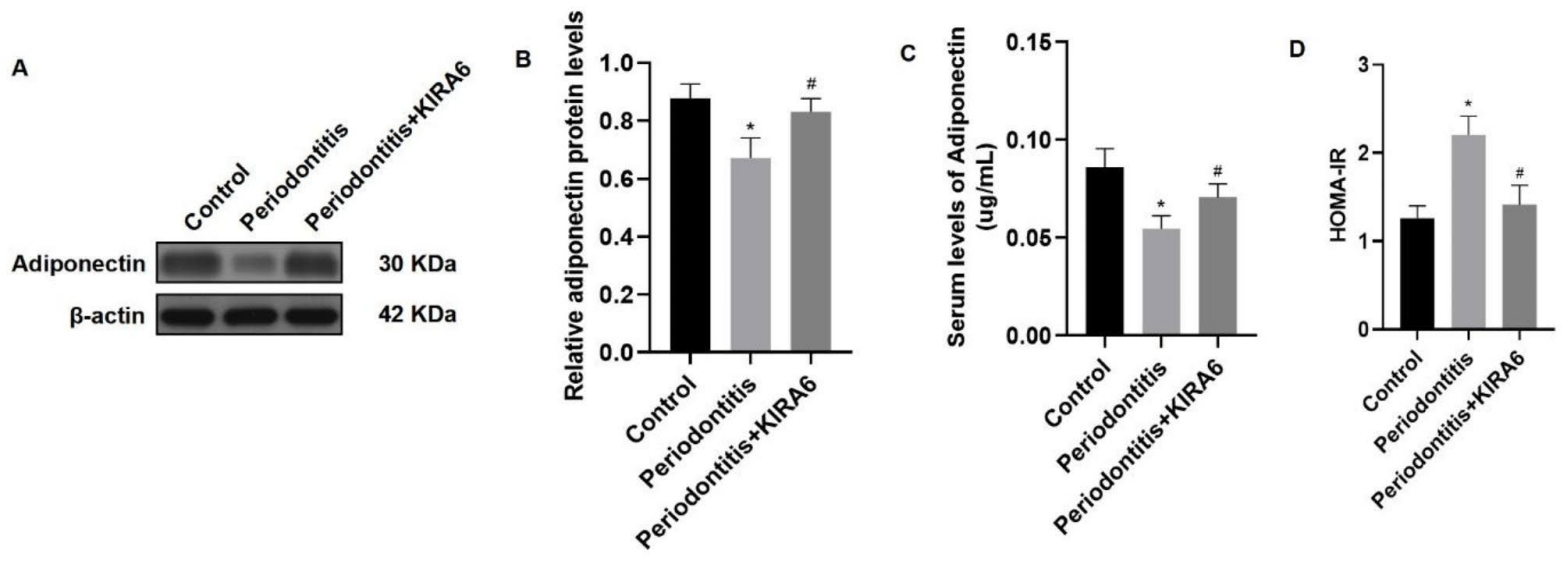
**Epididymal adipose tissue injection of KIRA6 improved hypoadiponectinemia and insulin resistance in rats with periodontitis.** (**A**) Western blotting image of adiponectin expression. (**B**) Adiponectin expression in the periodontitis + KIRA6 group was higher than that in the periodontitis group. (**C**) The serum adiponectin level in the periodontitis + KIRA6 group was higher than that in the periodontitis group. (**D**) HOMA-IR in the periodontitis + KIRA6 group was lower than that in the periodontitis group. The samples derive from the same experiment and that blots were processed in parallel. The full-length blots are presented in Supplementary File. 1 Fig. 4S. Data are presented as the mean ± SD. *P < 0.05 vs. the control group, #*P* < 0.05 vs. the periodontitis group

Adiponectin level in epididymal adipose tissue was detected using western blotting (Fig. 5A). The adiponectin expression in the periodontitis + KIRA6 group was higher than that in the periodontitis group (*P* < 0.05) (Fig. 5B). ELISA analysis showed that compared with that in the periodontitis group, the serum adiponectin level in the periodontitis + KIRA6 group was increased (*P* < 0.05) (Fig. 5C). HOMA-IR was decreased in the periodontitis + KIRA6 group compared with the periodontitis group (*P* < 0.05) (Fig. 5D). The results indicated that hypoadiponectinemia and insulin resistance in periodontitis rats were improved after inhibiting IRE1α in visceral adipose tissue.

## Discussion

Periodontitis is associated with the development of various systemic diseases, especially insulin resistance and T2D [11]. Hypoadiponectinemia is an important cause of insulin resistance [17]. Therefore, hypoadiponectinemia may be a key stage in the occurrence and development of T2D that is mediated or aggravated by periodontitis. However, the potential mechanisms by which periodontitis induces hypoadiponectinemia are unclear. Therefore, we constructed an in vivo model of periodontitis in rats and an in vitro model of *P.g*-LPS-induced cells, found that ERS in visceral adipocytes is involved in periodontitis-induced hypoadiponectinemia and revealed the primary mechanism involved.

Periodontal ligation is commonly used to establish experimental periodontitis in animal models. The placement of ligation silk leads to disordered subgingival flora to increase the abundance of gram-negative anaerobic bacteria such as *P. gingivalis*, and a large amount of LPS is secreted, which induces immune damage and leads to the destruction of periodontal tissue [38–40]. In this study, periodontal ligation caused significant inflammatory destruction of the gingival tissues and resorption of alveolar bone. After 3 months of periodontitis induction, we detected a significant decrease in serum adiponectin levels, known as hypoadiponectinemia, in the rats with periodontitis, which is consistent with epidemiological surveys [17]. These results proved that we successfully simulated the periodontitis-induced hypoadiponectinemic state in rats. Interestingly, we also observed increased fasting serum insulin levels in rats, and HOMA-IR was significantly higher in the periodontitis group than in the control group but was inversely correlated with serum adiponectin levels. This is consistent with clinical reports that hypoadiponectinemia can lead to insulin resistance [10].

In the context of periodontitis, virulence factors secreted by various periodontal pathogenic bacteria from the site of periodontal infection may enter the systemic circulation and play a positive regulatory role in the pathological changes of various tissues and cells far from the mouth [22, 41, 42]. *P.g*-LPS is currently recognized as the virulence factor of periodontitis and is also the most commonly stimulating factor for periodontitis research in vitro. Our previous studies used *P.g*-LPS to stimulate visceral adipocytes. We found that *P.g*-LPS reduced adiponectin secretion and led to impaired insulin signaling [30, 43]. Therefore, in this experiment, we constructed a *P.g*-LPS-stimulated visceral adipocytes model in vitro [30]. According to our previous studies, visceral adipocytes were stimulated by 100 ng/mL *P.g*-LPS at various times in vitro. This concentration is commonly used in the range of concentrations (0 to 10,000 ng/mL) reported for *P.g*-LPS in in vitro and in vivo studies [44, 45]. Based on in vitro and in vivo studies, we found that periodontitis could reduce adiponectin protein expression in rat visceral adipocytes and that adiponectin expression in visceral adipocytes stimulated by *P.g*-LPS was also reduced in a time-dependent manner.

ERS is associated with adiponectin secretion. To observe whether ERS occurs in visceral adipose tissue of rats with periodontitis, we detected the ERS-associated proteins GRP78, IRE1α, PERK and ATF6. The results showed that the transcription and protein levels of GRP78 and IRE1α in the periodontitis group were significantly higher than those in the control group, and PERK and ATF6 were not changed. At the same time, the p-IRE1α/IRE1α ratios were increased in the periodontitis group compared with those of the control group, but p-PERK/PERK did not change. Based on the results of animal experiments, we tested the protein levels of GRP78, p-IRE1α and IRE1α in visceral adipocytes stimulated by *P.g*-LPS and showed that GRP78 protein levels and the p-IRE1α/IRE1α ratio increased in a time-dependent manner. Under physiological conditions, GRP78 resides in the ER and closely binds to three transmembrane proteins. In response to external stimuli, GRP78 separates from transmembrane proteins and activates the unfolded protein response (UPR) to produce ERS [31]. Therefore, GRP78 is considered a specific marker for the early development of ERS. Consistent with our results, the stimulation of human periodontal ligament cells with *P.g*-LPS produced ERS, mainly represented by increased expression of GRP78 protein [46]. In addition, *P.g*-LPS stimulation of THP-1 human monocytic cells can also lead to upregulation of GRP78 protein expression [47]. These results indicated that GRP78 was sensitive to *P.g*-LPS stimulation and can be used as a classical marker of ERS induction by *P.g*-LPS in different cells. Elevated GRP78 dissociates itself from IRE1α and activates IRE1α [48]. Activated IRE1α is phosphorylated to form a dimer, further activating downstream signalling pathways [49]. Thus, elevated p-IRE1α expression represents a further enhancement of ERS. Similar to our results, stimulation of the mouse trachea with *Escherichia coli* LPS (*E. coli*-LPS) led to upregulation of GRP78 and p-IRE1α protein levels in lung tissue and the development of ERS [48]. LPS upregulated the key ERS proteins GRP78 and IRE1α, and promoted pathological changes in tissues. Our results confirm that *P.g*-LPS-induced periodontitis causes ERS in visceral adipocytes by upregulating the expression of GRP78 and p-IRE1α.

Studies have found that ERS in visceral adipocytes leads to decreased adiponectin expression through different mechanisms. For example, obesity-induced ERS in visceral adipocytes reduced adiponectin expression mainly through the inhibition of transcription activity by ER chaperone protein disulphide-isomerase A4 (PDIA4) [50]. The small extracellular vesicles (SEVs) released by ischemic/reperfused cardiomyocytes induce ERS in visceral adipocytes and downregulated ER degradation enhancing alpha-mannosidase like protein 3 (EDEM3), resulting in decreased adiponectin gene and protein expression [51]. However, whether *P.g*-LPS-mediated upregulation of IRE1α is a novel target for regulating adiponectin expression in visceral adipocytes has not been reported. This study used lentivirus transfection to construct a visceral adipocytes model with IRE1α overexpression. The results showed that IRE1α overexpression downregulated the transcription and protein levels of adiponectin. When accompanied by *P.g*-LPS stimulation, adiponectin was more significantly downregulated at both the transcriptional and protein levels. Conversely, transfection of visceral adipocytes by inhibiting IRE1α lentivirus led to elevated transcription and protein levels of adiponectin in adipocytes. Moreover, silencing IRE1α rescued the reduction in the transcription and protein levels of adiponectin in visceral adipocytes induced by *P.g***-**LPS stimulation. These results indicate that IRE1α is a downstream signalling molecule of *P.g-*LPS and plays a key role in regulating adiponectin synthesis in visceral adipocytes. Interestingly, although both *E. coli*- LPS and *P. g*-LPS caused visceral adipocytes dysfunction, the former did not cause reduced adiponectin secretion in visceral adipocytes [27]. It is well known that *E. coli*- LPS and *P. g*-LPS belong to the pathogen-associated molecular pattern (PAMP) [52]. They can bind to TLRs on the surface of visceral adipocytes to activate innate immune responses, thus leading to visceral adipocytes dysfunction [53]. The downregulation of adiponectin expression by *P.g*-LPS may not occur through the TLR-mediated innate immune response. The ERS signalling pathway is another important pathway by which *P.g*-LPS regulates the function of visceral adipocytes in addition to the innate immune response.

Contrary to the results of this study, scholars reported that elevated IRE1α in visceral adipocytes stimulated by insulin led to increased adiponectin expression, thereby promoting glucose uptake [34]. This implies that IRE1α is a key protein regulating adiponectin expression in visceral adipocytes, but the downstream signalling pathways may be different. Under different extracellular stimuli, IRE1α and its specific downstream signalling pathways are activated, thus affecting the expression of adiponectin to meet metabolic requirements. However, our study failed to clarify the downstream signalling molecules of IRE1α that are responsible for the decreased adiponectin expression after *P.g-*LPS stimulation. These molecules need deep exploration in future studies. To further verify that IRE1α-mediated ERS is the cause of hypoadiponectinemia induced by periodontitis, we used local silk ligation around the bilateral maxillary second molars and injected the IRE1α inhibitor KIRA6 into epididymal adipose tissue. Three months later, the serum adiponectin level in the periodontitis + KIRA6 group was significantly higher than that in the periodontitis group, and insulin resistance was relieved. Furthermore, the adiponectin protein level in visceral adipocytes in the periodontitis + KIRA6 group was also higher than that in the periodontitis group, consistent with the serological observations. These results suggest that inhibiting IRE1α in visceral adipocytes can improve periodontitis-induced hypoadiponectinemia. To our knowledge, this is the first demonstration of a potential mechanism between periodontitis and hypoadiponectinemia by an IRE1α inhibitor in rats.

In conclusion, this study demonstrated for the first time that periodontitis and its toxic product, *P.g-*LPS, can induce hypoadiponectinemia through IRE1α-mediated ERS in visceral adipocytes in vitro and in vivo. In the future, it is necessary to further clarify the relevant regulatory mechanism using visceral adipocyte-specific gene knockout IRE1α models. This study provides a theoretical basis for the clinical prevention and treatment of periodontitis-induced hypoadiponectinemia and associated systemic diseases.

### Electronic supplementary material

Below is the link to the electronic supplementary material.

Supplementary Material 1

Supplementary Material 2

## Data Availability

The datasets used and/or analyzed during the current study are available from corresponding author on reasonable request.

## References

[1] Fang H, Judd R (2018). Adiponectin regulation and function. Compr Physiol.

[2] da Silva Rosa SC, Liu M, Sweeney G (2021). Adiponectin synthesis, secretion and extravasation from circulation to interstitial space. Physiol (Bethesda).

[3] Ganesh V, Palem MM (2022). Adiponectin can be an early predictable marker for type 2 Diabetes Mellitus and Nephropathy. Cureus.

[4] Yamauchi T, Nio Y, Maki T, Kobayashi M, Takazawa T, Iwabu M, Okada-Iwabu M, Kawamoto S, Kubota N, Kubota T (2007). Targeted disruption of AdipoR1 and AdipoR2 causes abrogation of adiponectin binding and metabolic actions. Nat Med.

[5] Erdogan S, Sezer S, Baser E, Gun-Eryilmaz O, Gungor T, Uysal S, Yilmaz FM (2013). Evaluating vaspin and adiponectin in postmenopausal women with endometrial cancer. Endocr Relat Cancer.

[6] Gu C, Qu Y, Zhang G, Sun L, Zhu Y, Ye D (2015). A single nucleotide polymorphism in ADIPOQ predicts biochemical recurrence after radical prostatectomy in localized Prostate cancer. Oncotarget.

[7] Macis D, Aristarco V, Johansson H, Guerrieri-Gonzaga A, Raimondi S, Lazzeroni M, Sestak I, Cuzick J, DeCensi A, Bonanni B, et al. A novel automated immunoassay platform to evaluate the association of adiponectin and leptin levels with breast cancer risk. Cancers (Basel). 2021;13(13). 10.3390/cancers13133303 PMC8268385 34209441

[8] Inamura K, Song M, Jung S, Nishihara R, Yamauchi M, Lochhead P, Qian ZR, Kim SA, Mima K, Sukawa Y, et al. Prediagnosis plasma adiponectin in relation to Colorectal Cancer Risk according to KRAS Mutation Status. J Natl Cancer Inst. 2016;108(4). 10.1093/jnci/djv363 PMC4668768 26598515

[9] Kalkman HO. An explanation for the Adiponectin Paradox. Pharmaceuticals (Basel). 2021;14(12). 10.3390/ph14121266 PMC8703455 34959666

[10] Liu Z, Liang S, Que S, Zhou L, Zheng S, Mardinoglu A (2018). Meta-analysis of Adiponectin as a biomarker for the detection of metabolic syndrome. Front Physiol.

[11] Yoshimoto M, Sakuma Y, Ogino J, Iwai R, Watanabe S, Inoue T, Takahashi H, Suzuki Y, Kinoshita D, Takemura K, et al. Sex differences in predictive factors for onset of type 2 diabetes in Japanese individuals: a 15-year follow-up study. J Diabetes Investig. 2022. 10.1111/jdi.13918 PMC9807159 36200977

[12] Xiong QY, Xiong CQ, Wang LZ, Gao JL. Effect of sidt2 gene on cell insulin resistance and its molecular mechanism. J Diabetes Res. 2020;4217607. 10.1155/2020/4217607 PMC7502120 32964053

[13] Fernandes I, Oliveira J, Pinho A, Carvalho E. The role of nutraceutical containing polyphenols in diabetes prevention. Metabolites. 2022;12(2). 10.3390/metabo12020184 PMC8878446 35208257

[14] Yamauchi T, Kamon J, Waki H, Terauchi Y, Kubota N, Hara K, Mori Y, Ide T, Murakami K, Tsuboyama-Kasaoka N (2001). The fat-derived hormone adiponectin reverses insulin resistance associated with both lipoatrophy and obesity. Nat Med.

[15] Moyce Gruber B, Cole L, Xiang B, Fonseca M, Klein J, Hatch G, Doucette C, Dolinsky V (2022). Adiponectin deficiency induces hepatic steatosis during pregnancy and gestational Diabetes in mice. Diabetologia.

[16] Sete MR, Lira Junior R, Fischer RG, Figueredo CM (2015). Serum adipokine levels and their relationship with fatty acids in patients with chronic periodontitis. Braz Dent J.

[17] Zhu J, Guo B, Gan X, Zhang L, He Y, Liu B, Chen X, Zhang S, Yu H (2017). Association of circulating leptin and adiponectin with periodontitis: a systematic review and meta-analysis. BMC Oral Health.

[18] Kinane DF, Stathopoulou PG, Papapanou PN (2017). Periodontal Diseases. Nat Rev Dis Primers.

[19] Jiang Y, Yang P, Li C, Lu Y, Kou Y, Liu H, Guo J, Li M. Periostin regulates LPS-induced apoptosis via Nrf2/HO-1 pathway in periodontal ligament fibroblasts. Oral Dis. 2022. 10.1111/odi.14189 35298860

[20] Chen MX, Zhong YJ, Dong QQ, Wong HM, Wen YF (2021). Global, regional, and national burden of severe periodontitis, 1990–2019: an analysis of the global burden of Disease Study 2019. J Clin Periodontol.

[21] Kim YT, Jeong J, Mun S, Yun K, Han K, Jeong SN (2022). Comparison of the oral microbial composition between healthy individuals and periodontitis patients in different oral sampling sites using 16S metagenome profiling. J Periodontal Implant Sci.

[22] Seyama M, Yoshida K, Yoshida K, Fujiwara N, Ono K, Eguchi T, Kawai H, Guo J, Weng Y, Haoze Y (2020). Outer membrane vesicles of Porphyromonas gingivalis attenuate insulin sensitivity by delivering gingipains to the liver. Biochim Biophys Acta Mol Basis Dis.

[23] Su Y, Ye L, Hu C, Zhang Y, Liu J, Shao L (2023). Periodontitis as a promoting factor of T2D: current evidence and mechanisms. Int J Oral Sci.

[24] Matsuo I, Kawamura N, Ohnuki Y, Suita K, Ishikawa M, Matsubara T, Mototani Y, Ito A, Hayakawa Y, Nariyama M (2022). Role of TLR4 signaling on Porphyromonas gingivalis LPS-induced cardiac dysfunction in mice. PLoS ONE.

[25] Blasco-Baque V, Garidou L, Pomie C, Escoula Q, Loubieres P, Le Gall-David S, Lemaitre M, Nicolas S, Klopp P, Waget A (2017). Periodontitis induced by Porphyromonas gingivalis drives periodontal microbiota dysbiosis and insulin resistance via an impaired adaptive immune response. Gut.

[26] Ding LY, Liang LZ, Zhao YX, Yang YN, Liu F, Ding QR, Luo LJ (2019). Porphyromonas gingivalis-derived lipopolysaccharide causes excessive hepatic lipid accumulation via activating NF-kappaB and JNK signaling pathways. Oral Dis.

[27] Le Sage F, Meilhac O, Gonthier MP (2017). Anti-inflammatory and antioxidant effects of polyphenols extracted from Antirhea borbonica medicinal plant on adipocytes exposed to Porphyromonas gingivalis and Escherichia coli lipopolysaccharides. Pharmacol Res.

[28] Kadowaki T, Yamauchi T, Kubota N, Hara K, Ueki K, Tobe K (2006). Adiponectin and adiponectin receptors in insulin resistance, Diabetes, and the metabolic syndrome. J Clin Invest.

[29] Brum R, Duarte P, Canto G, Flores-Mir C, Benfatti C, Porporatti A, Zimmermann G (2020). Biomarkers in biological fluids in adults with periodontitis and/or obesity: a meta-analysis. J Indian Soc Periodontology.

[30] Lv YT, Zeng JJ, Lu JY, Zhang XY, Xu PP, Su Y (2021). Porphyromonas gingivalis lipopolysaccharide (Pg-LPS) influences adipocytes injuries through triggering XBP1 and activating mitochondria-mediated apoptosis. Adipocyte.

[31] Liu L, Zhao M, Jin X, Ney G, Yang KB, Peng F, Cao J, Iwawaki T, Del Valle J, Chen X (2019). Adaptive endoplasmic reticulum stress signalling via IRE1alpha-XBP1 preserves self-renewal of haematopoietic and pre-leukaemic stem cells. Nat Cell Biol.

[32] Guo Q, Jin S, Hu H, Zhou Y, Yan Y, Zong H, Wang Y, He H, Oh Y, Liu C (2017). Hypoxia in 3T3-L1 adipocytes suppresses adiponectin expression via the PERK and IRE1 unfolded protein response. Biochem Biophys Res Commun.

[33] Hosogai N, Fukuhara A, Oshima K, Miyata Y, Tanaka S, Segawa K, Furukawa S, Tochino Y, Komuro R, Matsuda M (2007). Adipose tissue hypoxia in obesity and its impact on adipocytokine dysregulation. Diabetes.

[34] Cho YM, Kim DH, Lee KH, Jeong SW, Kwon OJ (2018). The IRE1alpha-XBP1s pathway promotes insulin-stimulated glucose uptake in adipocytes by increasing PPARgamma activity. Exp Mol Med.

[35] Zeng J, Jia N, Ji C, Zhong S, Chai Q, Zou C, Chen L (2022). Plaque control alleviated renal damage that was aggravated by experimental periodontitis in obese rats. Oral Dis.

[36] Yi S, Chen K, Zhang L, Shi W, Zhang Y, Niu S, Jia M, Cong B, Li Y (2019). Endoplasmic reticulum stress is involved in stress-Induced hypothalamic neuronal Injury in rats via the PERK-ATF4-CHOP and IRE1-ASK1-JNK pathways. Front Cell Neurosci.

[37] Huang X, Yu T, Ma C, Wang Y, Xie B, Xuan D, Zhang J (2016). Macrophages play a key role in the obesity-Induced Periodontal Innate Immune Dysfunction via nucleotide-binding oligomerization domain-like receptor protein 3 pathway. J Periodontol.

[38] Kirst ME, Li EC, Alfant B, Chi YY, Walker C, Magnusson I, Wang GP (2015). Dysbiosis and alterations in predicted functions of the subgingival microbiome in chronic periodontitis. Appl Environ Microbiol.

[39] Patini R, Staderini E, Lajolo C, Lopetuso L, Mohammed H, Rimondini L, Rocchetti V, Franceschi F, Cordaro M, Gallenzi P (2018). Relationship between oral microbiota and periodontal Disease: a systematic review. Eur Rev Med Pharmacol Sci.

[40] Wang GP (2015). Defining functional signatures of dysbiosis in periodontitis progression. Genome Med.

[41] Ilievski V, Toth PT, Valyi-Nagy K, Valyi-Nagy T, Green SJ, Marattil RS, Aljewari HW, Wicksteed B, O’Brien-Simpson NM, Reynolds EC (2020). Identification of a periodontal pathogen and bihormonal cells in pancreatic islets of humans and a mouse model of periodontitis. Sci Rep.

[42] Yamazaki K, Kato T, Tsuboi Y, Miyauchi E, Suda W, Sato K, Nakajima M, Yokoji-Takeuchi M, Yamada-Hara M, Tsuzuno T (2021). Oral Pathobiont-Induced changes in Gut Microbiota aggravate the Pathology of nonalcoholic fatty Liver Disease in mice. Front Immunol.

[43] Lu JY, Wu QQ, Chen YY, Ye LL, Su Y (2022). Mechanism of * Porphyomonas gingivalis * -lipopolysaccharide in regulating the insulin signaling pathway in adipocytes via X-box binding protein 1. West China Journal of Stomatology.

[44] Gugliandolo E, Fusco R, D’Amico R, Militi A, Oteri G, Wallace JL, Di Paola R, Cuzzocrea S (2018). Anti-inflammatory effect of ATB-352, a H2S -releasing ketoprofen derivative, on lipopolysaccharide-induced periodontitis in rats. Pharmacol Res.

[45] Tang J, Wu T, Xiong J, Su Y, Zhang C, Wang S, Tang Z, Liu Y (2015). Porphyromonas gingivalis lipopolysaccharides regulate functions of bone marrow mesenchymal stem cells. Cell Prolif.

[46] Bai Y, Wei Y, Wu L, Wei J, Wang X, Bai Y (2016). C/EBP beta mediates endoplasmic reticulum stress regulated inflammatory response and extracellular matrix degradation in LPS-Stimulated Human Periodontal Ligament cells. Int J Mol Sci.

[47] Saba JA, McComb ME, Potts DL, Costello CE, Amar S (2007). Proteomic mapping of stimulus-specific signaling pathways involved in THP-1 cells exposed to Porphyromonas gingivalis or its purified components. J Proteome Res.

[48] Chen M, Li J, Liu X, Song Z, Han S, Shi R, Zhang X (2021). Chrysin prevents lipopolysaccharide-induced acute lung injury in mice by suppressing the IRE1alpha/TXNIP/NLRP3 pathway. Pulm Pharmacol Ther.

[49] Sun S, Shi G, Sha H, Ji Y, Han X, Shu X, Ma H, Inoue T, Gao B, Kim H (2015). IRE1alpha is an endogenous substrate of endoplasmic-reticulum-associated degradation. Nat Cell Biol.

[50] Su SC, Chien CY, Chen YC, Chiang CF, Lin FH, Kuo FC, Huang CL, Li PF, Liu JS, Lu CH (2022). PDIA4, a novel ER stress chaperone, modulates adiponectin expression and inflammation in adipose tissue. BioFactors.

[51] Gan L, Liu D, Xie D, Bond Lau W, Liu J, Christopher TA, Lopez B, Liu L, Hu H, Yao P (2022). Ischemic heart-derived small extracellular vesicles impair adipocyte function. Circ Res.

[52] Harm S, Schildbock C, Strobl K, Hartmann J (2021). An in vitro study on factors affecting endotoxin neutralization in human plasma using the Limulus amebocyte lysate test. Sci Rep.

[53] Le Sage F, Meilhac O, Gonthier MP (2017). Porphyromonas gingivalis lipopolysaccharide induces pro-inflammatory adipokine secretion and oxidative stress by regulating toll-like receptor-mediated signaling pathways and redox enzymes in adipocytes. Mol Cell Endocrinol.

