# Supplementary material for: Experimental periodontitis induced hypoadiponectinemia by IRE1α-mediated endoplasmic reticulum stress in adipocytes
Source: BMC Oral Health. 2023 Dec 21;23:1032. doi: 10.1186/s12903-023-03758-6 (PMC10740306; doi:10.1186/s12903-023-03758-6)
Supplement: Supplementary file 1 — Supplementary Material 1 [file 12903_2023_3758_MOESM1_ESM.pdf]

Fig. 2

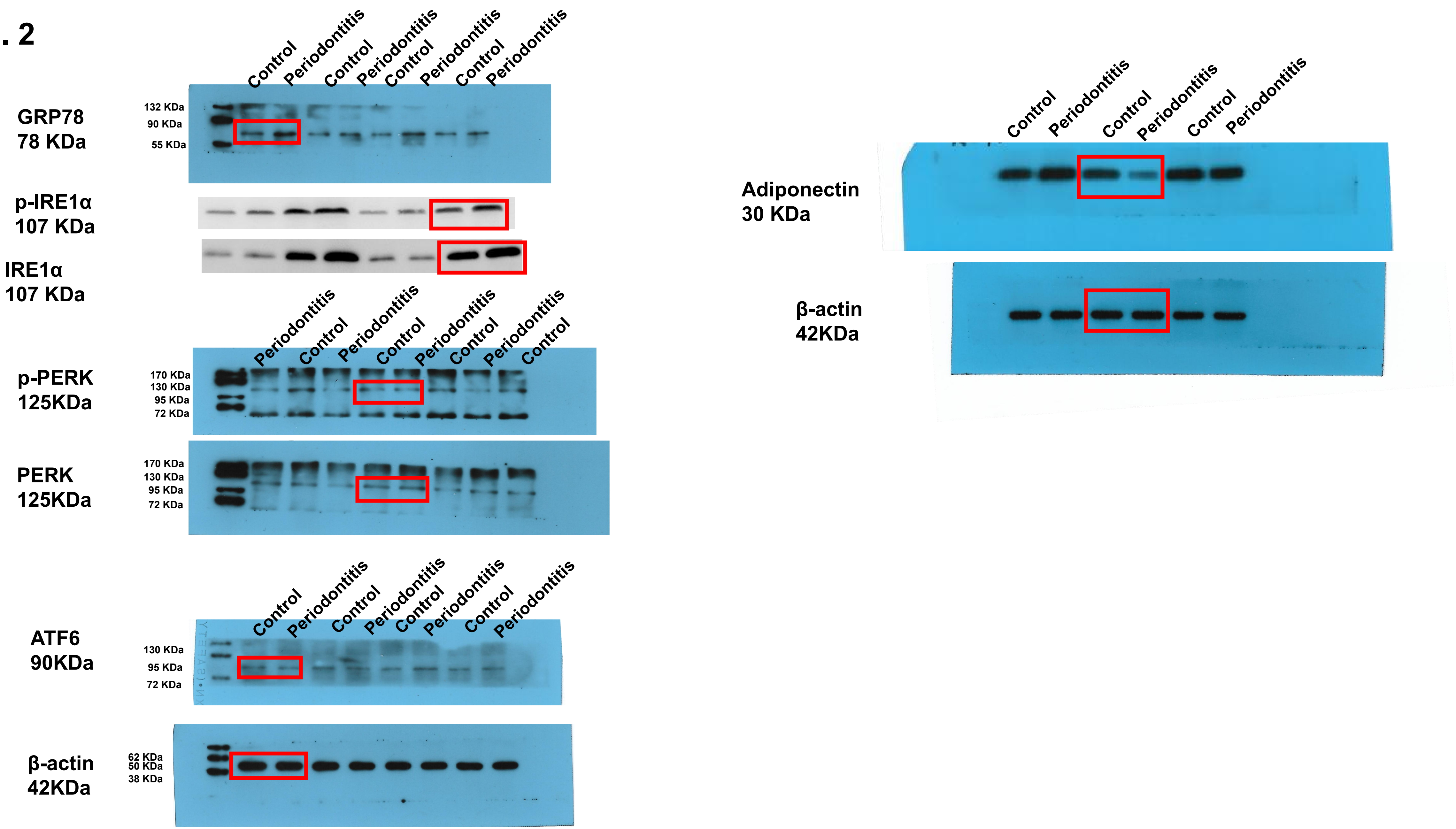

Fig. 1S. The original Western Blot images for GRP78, p-IRE1α, IRE1α, p-PERK, PERK, ATF6, adiponectin and β-actin in epididymal adipose tissue. The red box represents the blots displayed in the manuscrip (left, related to Fig. 2B; right, related to Fig. 2E).

Fig. 3

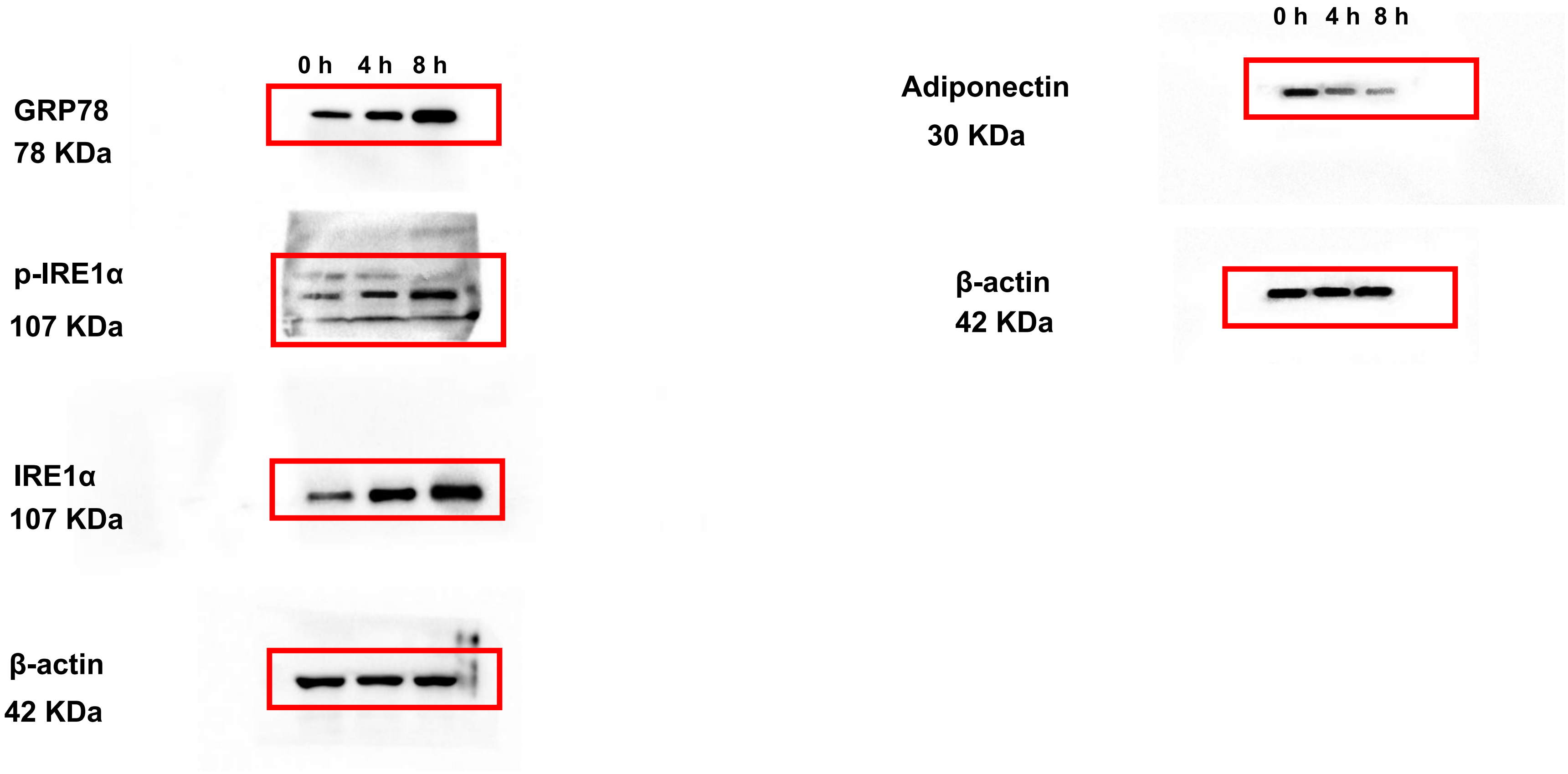

Fig. 2S. The original Western Blot images for GRP78, p-IRE1α, IRE1α, adiponectin and β-actin in visceral adipocytes (left, related to Fig. 3C; right, related to Fig. 3E).

Fig. 4

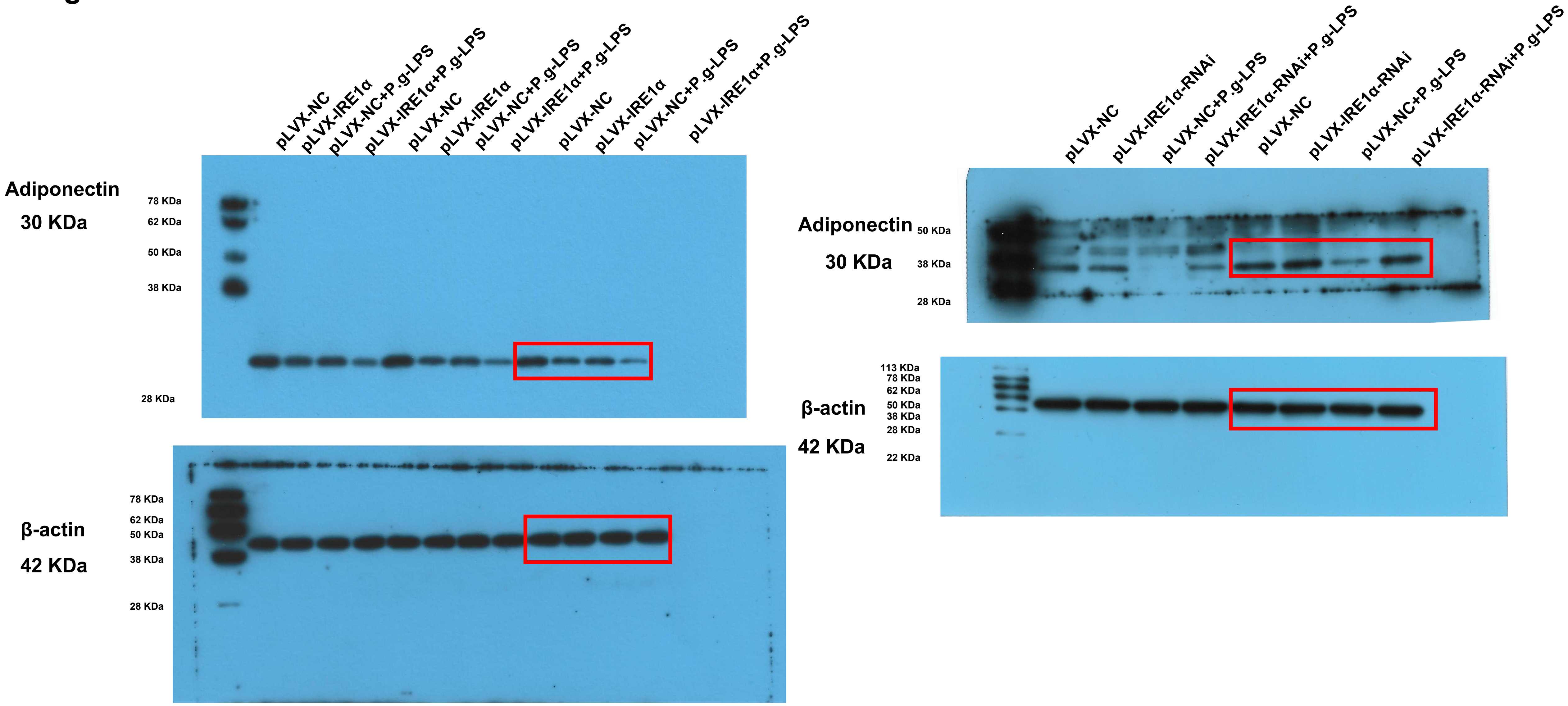

Fig. 3S. The original Western Blot images for adiponectin and β-actin in visceral adipocytes and the red box represents the blots displayed in the manuscript (left, related to Fig. 4B; right, related to Fig. 4E).

Fig. 5

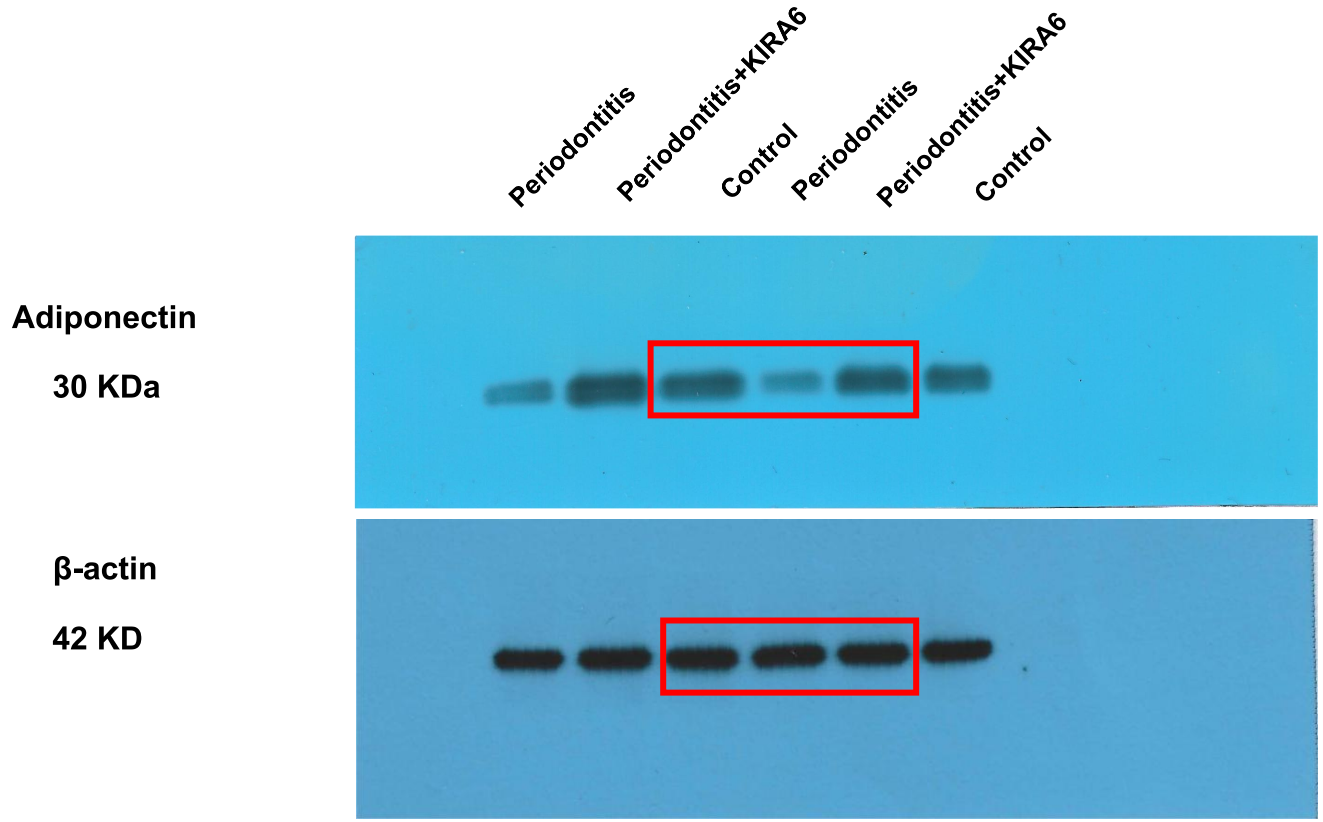

Fig. 4S. The original Western Blot images for adiponectin and β-actin in epididymal adipose tissue and the red box represents the blots displayed in the manuscript (related to Fig. 5A).
