# Supplementary material for: Experimental periodontitis induced hypoadiponectinemia by IRE1α-mediated endoplasmic reticulum stress in adipocytes
Source: BMC Oral Health. 2023 Dec 21;23:1032. doi: 10.1186/s12903-023-03758-6 (PMC10740306; doi:10.1186/s12903-023-03758-6)
Supplement: Supplementary file 2 — Supplementary Material 2 [file 12903_2023_3758_MOESM2_ESM.pdf]

Fig. 4

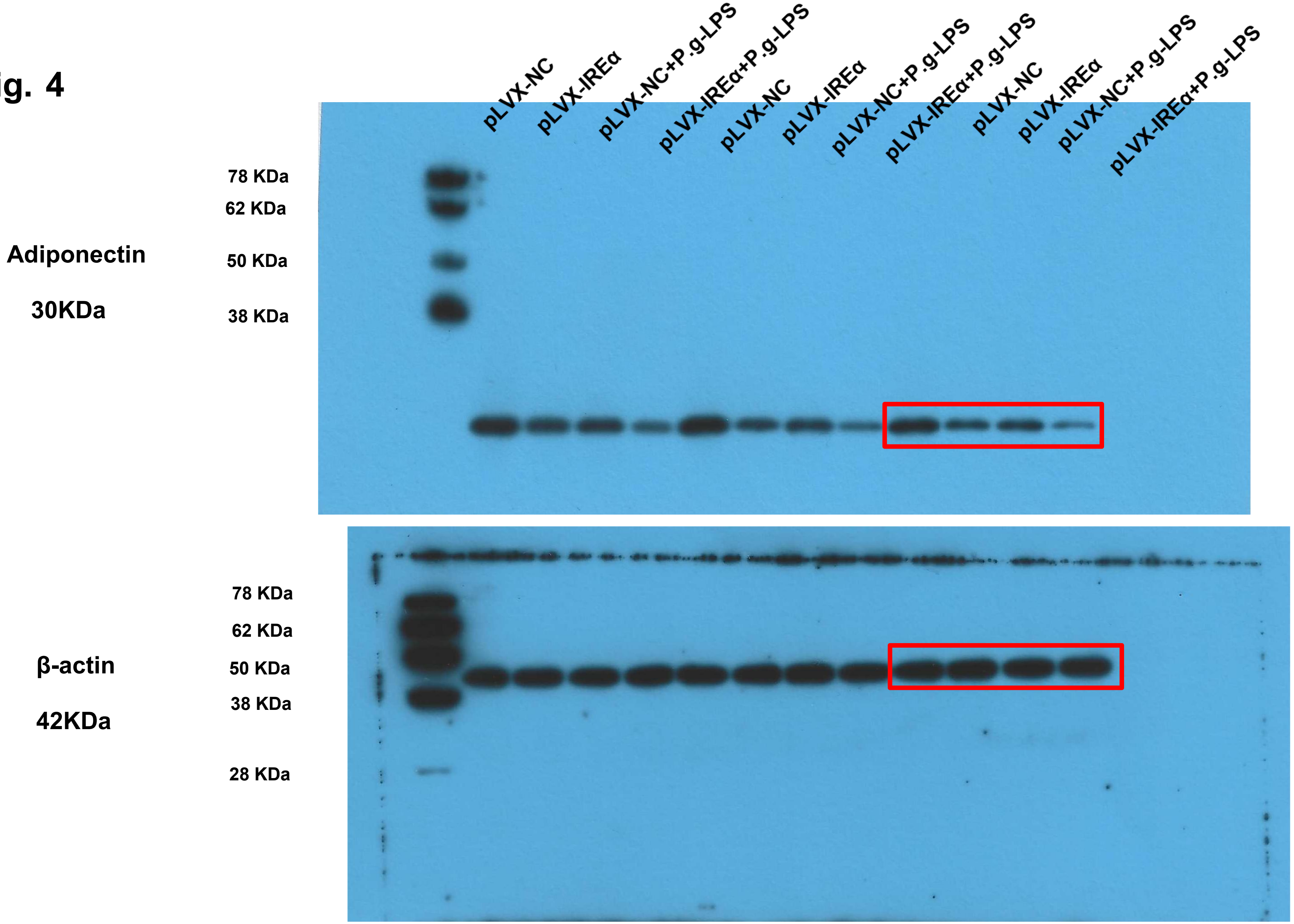

Fig. 5S. The experiment was redone once again. The original Western Blot images for adiponectin and β-actin in visceral adipocytes and the red box represents the blots displayed in the manuscrip (related to Fig. 4B) .

Supplementary Materials

1. The expression levels of TNF-α in rat epididymal adipose tissue

The expression levels of TNF-α in rat epididymal adipose tissue were significantly higher in the periodontitis group than in the control group (*P*<0.001) (Fig. S6 A-B).

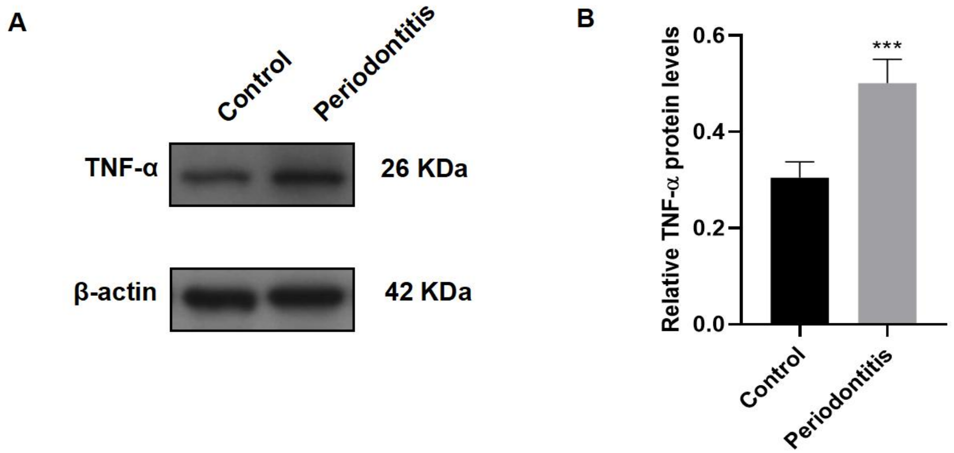

Fig. S6 Analysis of changes in TNF-α expression in epidiymal adipose tissue by periodontitis. (A) Western blotting image of TNF-α expression. (B) The TNF-α expression level in the periodontitis group was significantly higher than that in the control group. Data are presented as the mean ± SD. ****P*<0.001.

2. The expression levels of TNF-α in visceral adipocytes

100 ng/mL *P.g*-LPS was used to stimulate visceral adipocytes for 0 and 4 h. The expression levels of TNF-α in visceral adipocytes were significantly higher in the 4 h group than in the 0 h group (*P*<0.01) (Fig. S7 A-B)

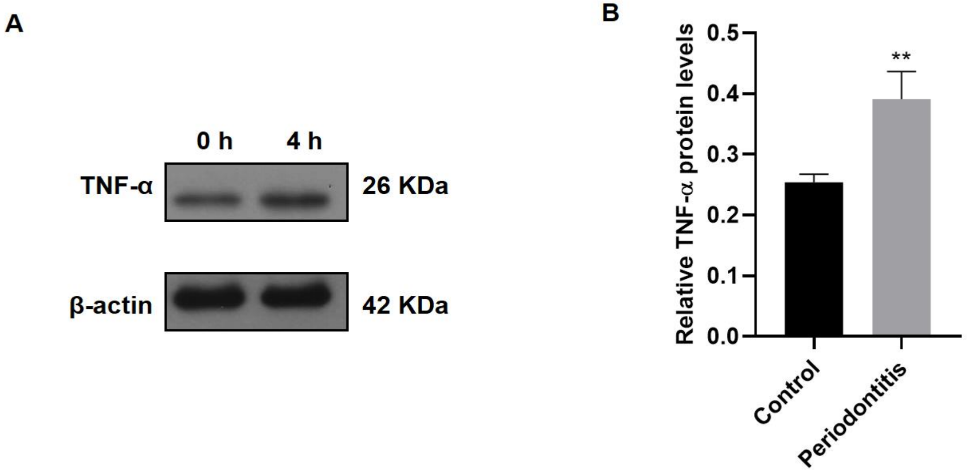

Fig. S7 Analysis of changes in TNF-α expression in visceral adipocytes by *P.g*-LPS stimulation. (A) Western blotting image of TNF-α expression. (B) The TNF-α expression level in the 4 h group was significantly higher than that in the 0 h group. Data are presented as the mean ± SD. ***P*<0.01.

Fig. S6

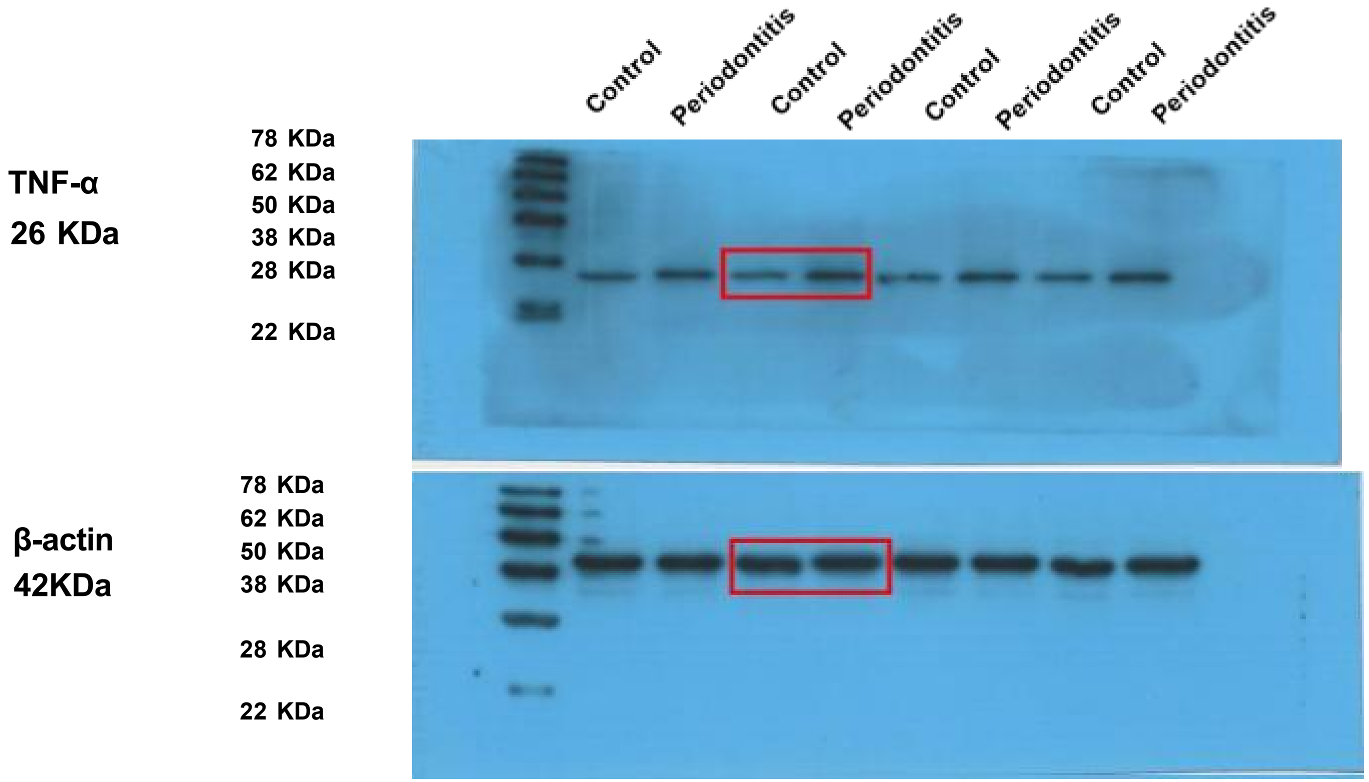

Fig. 8S. The original Western Blot images for TNF-α and β-actin in epididymal adipose tissue. The red box represents the blots displayed in the Supplementary Materials (related to Fig. 6S A).

Fig. 7S

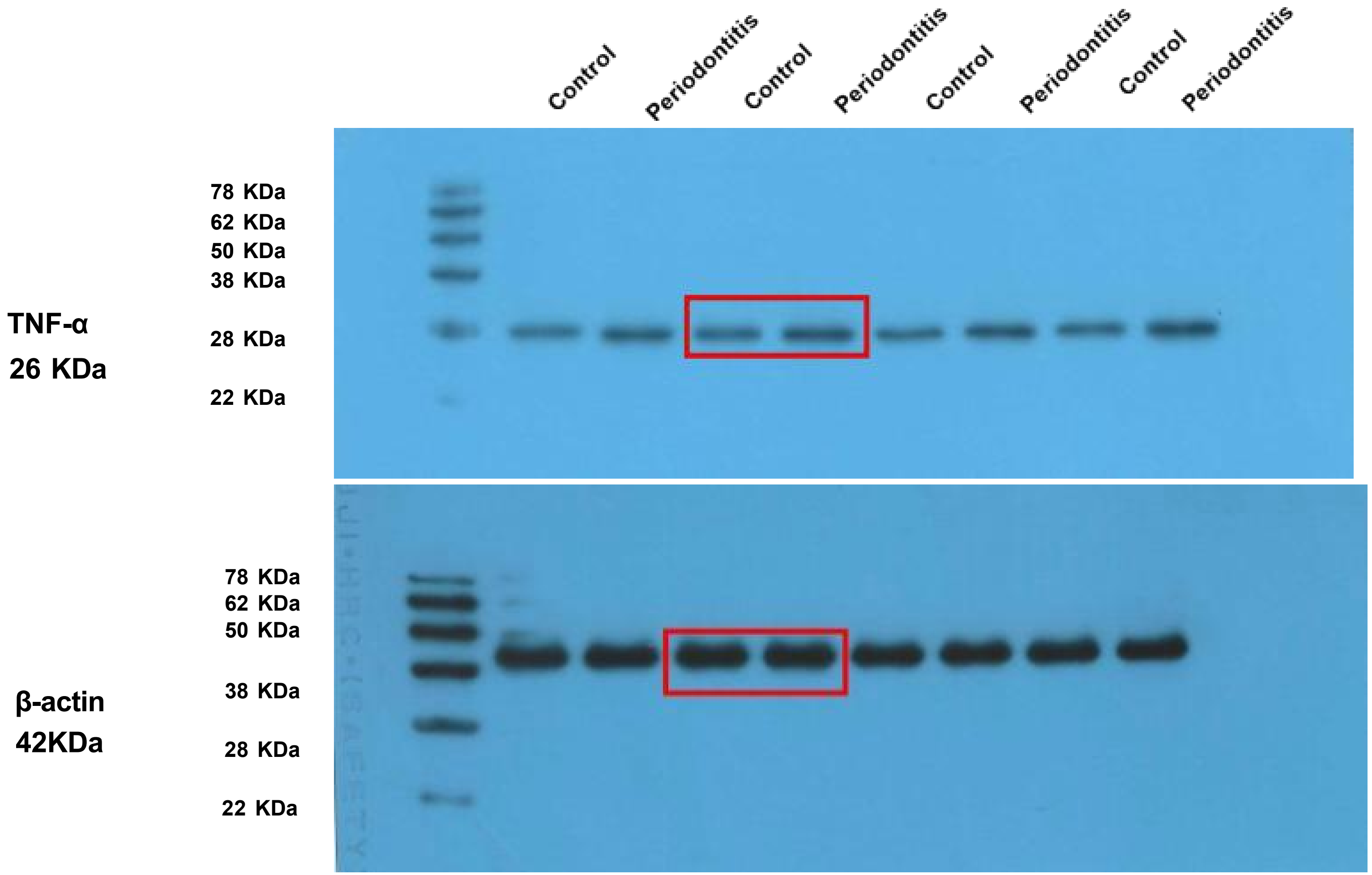

Fig. 9S. The original Western Blot images for TNF-α and β-actin in viscera adipocytes. The red box represents the blots displayed in the Supplementary Materials (related to Fig. 7S A).

Fig. 2

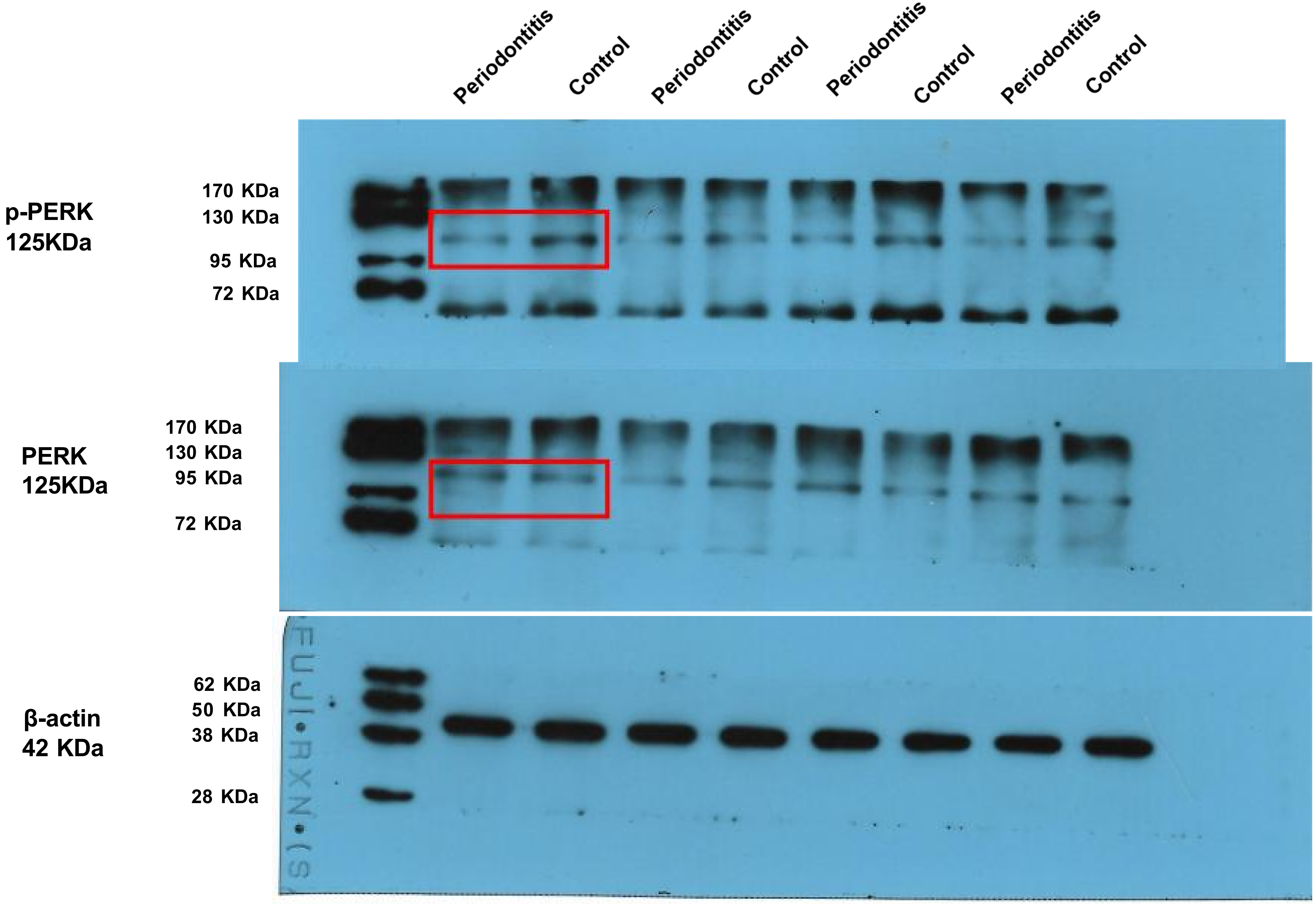

Fig. 10S. The original Western Blot images for p-PERK, PERK and β-actin in epididymal adipose tissue. The red box represents the blots displayed in the manuscrip (related to Fig. 2B).
